# Including a spatial predictive process in band recovery models improves inference for Lincoln estimates of animal abundance

**DOI:** 10.1002/ece3.9444

**Published:** 2022-10-27

**Authors:** Matthew Gonnerman, Daniel W. Linden, Stephanie A. Shea, Kelsey Sullivan, Pauline Kamath, Erik Blomberg

**Affiliations:** ^1^ Department of Wildlife Fisheries and Conservation Biology University of Maine Orono Maine USA; ^2^ Greater Atlantic Regional Fisheries Office NOAA National Marine Fisheries Service Gloucester Massachusetts USA; ^3^ School of Food and Agriculture University of Maine Orono Maine USA; ^4^ Maine Department of Inland Fisheries and Wildlife Bangor Maine USA

**Keywords:** band recovery, integrated population model, Lincoln estimator, spatial heterogeneity, spatial predictive process

## Abstract

Abundance estimation is a critical component of conservation planning, particularly for exploited species where managers set regulations to restrict harvest based on current population size. An increasingly common approach for abundance estimation is through integrated population modeling (IPM), which uses multiple data sources in a joint likelihood to estimate abundance and additional demographic parameters. Lincoln estimators are one commonly used IPM component for harvested species, which combine information on the rate and total number of individuals harvested within an integrated band‐recovery framework to estimate abundance at large scales. A major assumption of the Lincoln estimator is that banding and recoveries are representative of the whole population, which may be violated if major sources of spatial heterogeneity in survival or harvest rates are not incorporated into the model. We developed an approach to account for spatial variation in harvest rates using a spatial predictive process, which we incorporated into a Lincoln estimator IPM. We simulated data under different configurations of sample sizes, harvest rates, and sources of spatial heterogeneity in harvest rate to assess potential model bias in parameter estimates. We then applied the model to data collected from a field study of wild turkeys (*Meleagris gallapavo*) to estimate local and statewide abundance in Maine, USA. We found that the band recovery model that incorporated a spatial predictive process consistently provided estimates of adult and juvenile abundance with low bias across a variety of spatial configurations of harvest rate and sampling intensities. When applied to data collected on wild turkeys, a model that did not incorporate spatial heterogeneity underestimated the harvest rate in some subregions. Consistent with simulation results, this led to overestimation of both local and statewide abundance. Our work demonstrates that a spatial predictive process is a viable mechanism to account for spatial variation in harvest rates and limit bias in abundance estimates. This approach could be extended to large‐scale band recovery data sets and has applicability for the estimation of population parameters in other ecological models as well.

## Graphical abstract

We developed an approach to account for spatial variation in harvest rates using a spatial predictive process, which we incorporated into a Lincoln estimator IPM. We simulated data to assess potential model bias, then applied the model to data collected from a field study of wild turkeys (Meleagris gallapavo) to estimate local and statewide abundance in Maine, USA. Our work demonstrates that a spatial predictive process is a viable mechanism to account for spatial variation in harvest rates and limit bias in abundance estimates.

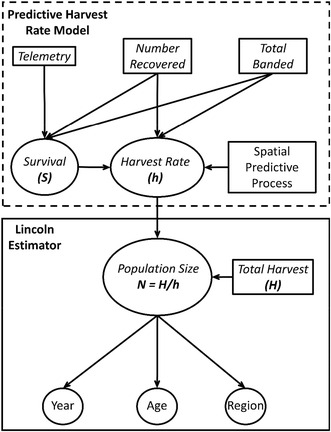

## 1 INTRODUCTION

Abundance estimation is a critical component of successful conservation planning (Thogmartin et al., 2006), particularly for exploited species where managers set quotas or other regulations to restrict harvest based on current population size (Nichols et al., 2007; Runge et al., 2009). If abundance is overestimated, liberal regulations may lead to a larger portion of the population being removed than intended, which can negatively impact long‐term stability (Johnson et al., 2012; Weinbaum et al., 2013). Alternatively, if population size is underestimated, harvest regulations may be set more restrictively than necessary, leading to underutilization of the resource, and reducing the opportunity for consumers. In either instance, there are benefits to identifying and implementing tools that estimate abundance as accurately as possible.

An increasingly common approach for abundance estimation is integrated population modeling (IPM; Chandler & Clark, 2014; Schaub & Abadi, 2011; Wilson et al., 2016), which uses multiple data sources in a joint likelihood to estimate abundance and additional demographic parameters. IPMs efficiently use data, provide a means of estimating uncertainty that is propagated among model parameters, and have the capacity to infer latent parameters for which data are not available. IPMs are versatile in the types of information they can incorporate, including capture‐mark‐recapture, point count, productivity, dead recovery, and telemetry data (Bled et al., 2017; Fay et al., 2019; Freeman & Crick, 2003; Horne et al., 2019; Lee et al., 2015), with the key requirement that one or more parameters are shared among the components of the IPM (Zipkin & Saunders, 2018).

Lincoln estimators (Lincoln, 1930) are increasingly used to estimate abundance at large scales (Alisauskas et al., 2009) by combining information on the rate and total number of individuals harvested; data that are typically obtained from band recoveries (Roberts et al., 2021) and hunter survey or harvest reporting. While historically underutilized, Lincoln estimators have been applied with great success in the management of multiple game species (Diefenbach et al., 2012; Hagen et al., 2014; Otis, 2006; Saunders et al., 2019), most notably for the harvest of waterfowl across North America (Alisauskas et al., 2014; Arnold et al., 2018). In an IPM framework, temporal dynamics in abundance can be represented in a state‐space approach, and additional data sources beyond those generated by harvest may be included to inform demographic parameters estimated by the model when available (Hostetler & Chandler, 2015; Tavecchia et al., 2009).

Despite the advantages of IPMs, violating assumptions of component models can lead to bias, both for parameters directly estimated from data and those being inferred indirectly (Riecke et al., 2019), making it important to both identify potential violations and implement reasonable solutions. A major assumption of the Lincoln estimator, shared by all band recovery models, is that banding and recoveries are representative of the whole population (Alisauskas et al., 2009), and this assumption will be violated if major sources of heterogeneity in survival or harvest rates are not incorporated into the model (Pollock & Raveling, 1982). Within a contiguous population, harvest rates may vary spatially according to variable harvest regulations, land access, weather, or land cover characteristics (Burke et al., 2019; Hansen et al., 1986; Norton et al., 2012). Similarly, survival may be linked to spatially varying factors like habitat, predation risk, or weather (Fleskes et al., 2007; Perkins et al., 1997; Tolon et al., 2009). Assumptions of constant harvest rate or survival may therefore be violated across large spatial scales, which will bias estimates at finer scales. When estimating parameters statewide and applying them to management objectives that are region‐specific, it is often unrealistic to assume that no heterogeneity exists among regions. Therefore, accounting for spatial heterogeneity is important to ensure accurate abundance estimates on which management will be based.

When incorporating spatial variation into models, accounting for multiple interacting factors can be difficult when each varies independently (Viana et al., 2013), and may be impractical to measure. It may be simpler to ignore specific causes of the spatial relationship, and instead take advantage of underlying spatial correlations in the data to map the spatial structure of parameters being estimated (Cressie, 2015). Locations that are closer together in space are more likely to be similar than those farther apart (Burrough, 1995), which can facilitate covariance functions to describe the spatially‐dynamic nature of a parameter. One such approach, spatial predictive processes (SPP; Banerjee et al., 2008), projects the underlying correlation among sampling sites onto a set of evenly spaced spatial knots distributed across an area of interest. SPP was initially intended as a dimension reduction approach to reduce computation requirements in Kriging for larger data sets (Banerjee et al., 2008), but the underlying framework has advantages beyond computational efficiency. For one, the covariance function does not require additional information beyond the locations of data, meaning that identifying and measuring explanatory covariates is unnecessary to represent underlying spatial heterogeneity in the process. Additionally, even spacing of spatial knots uniformly covers the area of interest, which is sometimes impractical for observed data when sampling depends on the presence of animals. Thus, using parameter estimates at evenly spaced knots may be more representative than those from sampling sites. Use of SPP is sparse within the ecological literature—although see examples for applying such an approach to estimating the spatial distribution of fisheries discards (Viana et al., 2013), avian communities (Jarzyna et al., 2014), or invasive plants (Latimer et al., 2009)—despite its broadly applicable approach for assessing spatial variation in vital rates, especially when causes of the spatial relationship are difficult to determine.

**FIGURE 1:**
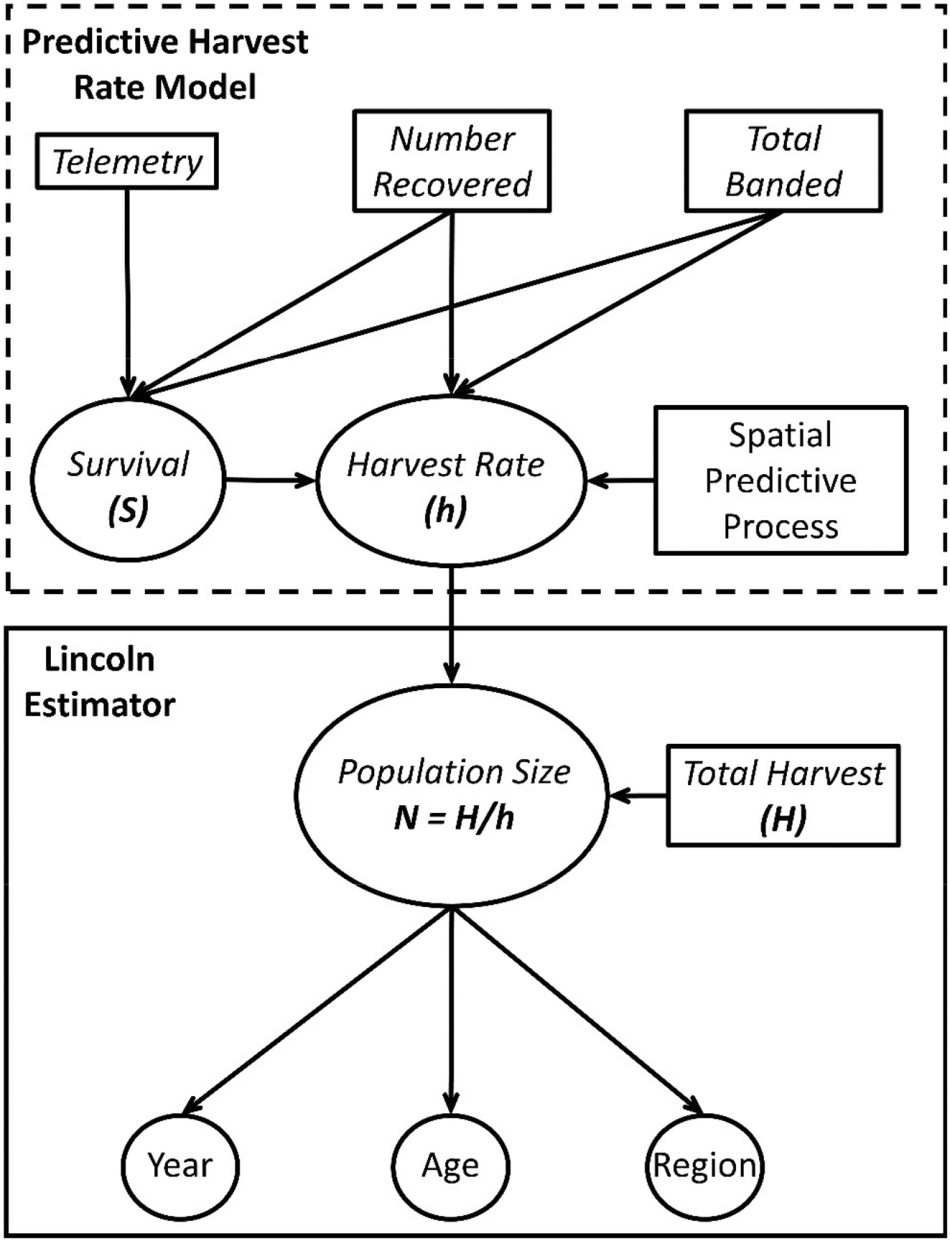
Band recovery, telemetry, and total harvest information in combination with a spatial predictive process can be used in a Lincoln estimator to produce estimates of harvest rate, survival, and abundance. The flow of data and parameter estimates through the integrated population model is depicted here by a directed acyclic graph.

Here, we develop and present an SPP approach for incorporating spatial variation in harvest rates into an IPM to derive robust estimates of wild turkey (*Meleagris gallapavo*; hereafter turkey) abundance. We integrated band recovery, telemetry, and total harvest data to estimate the region‐specific abundance of a two‐aged (adult and juvenile), harvested population at the beginning of the hunting season (Figure 1). Band recoveries were used to estimate harvest rate and survival rate under a modified Brownie parameterization of the dead recovery model (Brownie, 1985), in which we incorporated an SPP (Banerjee et al., 2008) to account for spatial variation in harvest rate among capture sites, and to allow estimation of harvest rates in areas where banding did not occur. To control for mortalities that occurred between banding and the beginning of the hunting season (Buderman et al., 2014), we linked survival in the band recovery model to a weekly survival rate estimated from telemetry data using a nest survival framework (Dinsmore et al., 2002). Final abundance estimates were generated using a Lincoln estimator within a state‐space approach (Alisauskas et al., 2014; Lincoln, 1930). We simulated data sets under different configurations of spatial variation in harvest rate and sampling characteristics to assess bias in parameter estimates and applied the model to data collected from a field study of wild turkeys to estimate abundance and inform harvest decisions across the state of Maine.

## 2 MATERIALS AND METHODS

### 2.1 Integrated population model

#### 2.1.1 Band recovery model

We used a modified version of the Brownie parameterization for dead recoveries (Brownie, 1985), where recovery rates were estimated as the combined probability that a bird was killed, retrieved, and reported by a hunter within a given hunting season. We considered recovery rate synonymous with “harvest rate” (*h*), which was estimated as a proportion of all mortalities (harvest plus nonharvest) that occurred throughout the year. We use the term “harvest” to refer to the total number of individuals harvested and reported to MDIFW (*H*). We assumed 100% reporting of harvested birds, although incomplete reporting could be incorporated with additional information on reporting rate. One assumption of all band recovery models is that no mortalities occur between capture and the beginning of the first hunting season postbanding, which is likely to be violated as time between the two events increases (Cooch et al., 2021). To better identify mortality that occurred between capture and the first hunting season, we separated each year within the conventional band recovery encounter history into two distinct and alternating occasion types, capture and the spring hunting season. The initial observation occurred during a capture occasion, and the terminal observation during a harvest occasion, resulting in two occasions per year. Survival was then differentiated according to three seasons within a year that corresponded with the intervals between recovery occasions; the period from the mid‐point of capture to the first day of harvest, the interval where harvest occurs (i.e., the hunting season), and the period from last day of the harvest season to the mid‐point of the following year's capture. For a given interval within the model, a Bernoulli random variable (ψ) was used to determine the probable latent survival state (*z*) of individual *i* at occasion *t*,

$$\mathrm{Pr}\left(z_{i,t}=1\right)∼\text{Bern}\left(\psi \right)$$

$$\psi =S_{i,t}\times w_{i,t–1}$$

where ψ was the probability of surviving all risks unrelated to reported harvests during the spring hunting season (*S*_*i,t*_) given that the bird was alive at the end of the previous occasion (*w*_*i,t*–1_ = 1). We modeled the probability of observed data using a Bernoulli random variable (γ),

$$\mathrm{Pr}\left(y_{i,t}=1\right)∼\text{Bern}\left(\gamma \right)$$

$$\gamma =h_{i,t}\times z_{i,t}$$

$$w_{i,t}=z_{i,t}–y_{i,t}$$

where *y* was the observed harvest of a banded individual and *h* was the probability a bird was harvested and reported, given that it survived all other mortality risks since the previous occasion (*z*_*i,t*_ = 1). Since harvests cannot occur during capture periods, we restricted *h* = 0 in capture occasions.

We modeled variation in *h* using a log–log‐linear model,

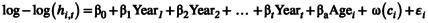




where β were coefficients that describe variation according to age of the individual (Juvenile, <1 year old, or Adult, >1 year old) and year of harvest, respectively.

To account for spatial variation in harvest rates, we included a mean‐zero SPP (ω[*c*]; Viana et al., 2013; see section 2.1.2) that depended on the capture location for all individuals, ***c*** *=* {*c*_*1*_, *c*_*2*_, …, *c*_*n*_}. Remaining variation was modeled by a nonspatial error term (*ε*_*i*_), where

$$\varepsilon_{i}∼N\left(0,\sigma^{2}\right)$$

#### 2.1.2 Spatial predictive process

Following the methods of Viana et al. (2013), we accounted for spatial variation in harvest rate by incorporating the mean‐zero SPP into the logit‐linear regression. We specified a set of evenly distributed spatial knots, c* *=* {c*_1_, c*_2_, …, c*_m_}, across the study area on which we defined a Gaussian process with exponential covariance,

$$\tilde{\omega }\left(c^{*}\right)∼\mathrm{GP}\left(0,\sigma_{s}^{2}\rho \left[c^{*},c^{*}|\;\phi \right]\right)$$

where *σ*_*s*_^2^ was the spatial random effect variance and ρ was an autocorrelation function with

$$\rho \left(c_{a},c_{b}\;|\;\phi \right)=\exp\left[-|d_{a,b}|/\phi \right]$$

where |*d*_*a,b*_| was the distance between locations *c*_*a*_ and *c*_*b*_, and ϕ determined the rate of decay in correlation as distance increased between locations. To project the Gaussian process from the knots back onto harvest locations, we used the correction for bias in *ε*_*i*_ proposed by Finley et al. (2009). Given a generic covariance function between two locations *C*(*c*_*a*_, *c*_*b*_ | ϕ) = *σ*_*s*_^2^ρ(*c*_*a*_, *c*_*b*_ | ϕ), we then defined ω(***s***) as

$$\omega \left(s\right)=C\left(c^{*},c\;|\;\phi \right)C{\left(c^{*},c^{*}\;|\;\phi \right)}^{-1}ῶ\left(c^{*}\right)+\varepsilon_{s}$$

$$\begin{array}{c} \varepsilon_{s}∼N\left(0,\mathrm{diag}\left(C\left(c,c|\phi \right)–C\left(c,c^{*}|\phi \right)C{\left(c^{*},c^{*}|\phi \right)}^{–1}C\left(c*,c|\phi \right)\right)\right) \end{array}$$

We then applied the covariance functions to the supplied sets of capture sites and spatial knots, which yielded site‐specific estimates of the harvest rate for each capture location.

#### 2.1.3 Weekly survival rate

Weekly survival rates (*s*) were estimated under a nest survival modeling framework (Dinsmore et al., 2002), in which we modeled whether an individual was observed alive since its previous telemetry observation (*x*) as a Bernoulli random variable (μ),

Pr(*x* = 1) ~ Bern(μ)

μ = *s* ^*k*^

where *s* was the probability of surviving 1 week and *k* was the number of weeks since an individual was last observed alive. We modeled variation in *s* using a log–log linear model (Ergon et al., 2018),

$$\log–\log\left(s\right)=\alpha_{0}+\alpha_{1}{\text{Region}}_{1}+\alpha_{2}{\text{Region}}_{2}+\ldots +\alpha_{r}{\text{Region}}_{r}+\alpha_{a}\mathrm{Age}+\alpha_{s}\text{Season}$$

where α represented individual, temporal, and spatial regression coefficients for categorical covariates age, season, and region, respectively. We used a covariate for the region an individual was captured to account for spatial variation in survival. To estimate survival from banding to harvest, we set *S* within the band recovery model equal to *s* exponentiated to reflect the relevant time period, such that

$$S_{i,t}=s^{n}$$

where *n* is the number of weeks between occasions within the band recovery model. For *n* following initial capture, we used the length of time from the time of marking to the beginning of the following harvest period. In subsequent occasions, we used the average number of weeks between the median date of capture and the first day of the hunting season to determine values for *n*. In practice this approach allowed us to accommodate the mortality of animals between capture and their first opportunity to be harvested, which cannot be reconciled in a standard band recovery framework (Buderman et al., 2014).

#### 2.1.4 State‐space abundance estimation

Abundance (*N*) was derived using a Lincoln estimator (Lincoln, 1930; Alisauskas et al., 2014) for each region and timestep as

$$N_{r,t}=\frac{H_{r,t}}{h_{r,t}}$$

where *r* was the region of the study area, and *t* was the year for which abundance was estimated. We linked abundance through time using a state‐space approach. *H*_*r,t*_ for each region were drawn from a binomial distribution

$$H_{r,t}∼\mathrm{Bin}\left({ĥ}_{r},\overset{\wedge }{N}{}_{r,t}\right)$$

where *ĥ*_*r*_ was the mean harvest rate across years for region *r* and was estimated as the average harvest rate at all spatial knots (h*_r_) within a region's boundaries, such that

$$\log–\log\left({h^{*}}_{r}\right)=\beta_{0}+\beta_{1}\mathrm{Age}+ῶ\left(c^{*}\right)$$

We assumed each region was closed to immigration and emigration, such that total abundance at the beginning of the hunting season (*N̂*_*a,r,t +* 1_) was equal to the number of adults that survived the previous year (*M*_*a,r,t)*_, combined with juveniles that survived from the previous year and graduated to adulthood (*M*_*j,r,t*_), each of which was drawn from binomial distributions

$$M_{a,r,t}∼\mathrm{Bin}\left(Q_{a,r},\overset{\wedge }{N}{}_{a,r,t}\right)$$

$$M_{j,r,t}∼\mathrm{Bin}\left(Q_{j,r},\overset{\wedge }{N}{}_{j,r,t}\right)$$

where *Q*_*r*_ was the total probability of survival, estimated as

$$Q_{r}=S\times \left(1–{ĥ}_{r}\right).$$

Harvest is often the most significant source of mortality for male turkeys and is dramatically greater than nonharvest mortality during the hunting season (Humberg et al., 2009); thus, we assumed that 1–*ĥ*_*r*_ represented the probability of surviving the spring hunting season by virtue of not being harvested and that nonharvest mortality during this interval was negligible. As we used categorical covariates to describe regional differences in survival and therefore could not directly estimate survival in regions in which we did not band, we estimated a global mean value for *S* for all regions by averaging survival in those regions where captures occurred. Juveniles were recruited into the population at the beginning of each hunting season at a per‐capita rate based on the number of adults alive in the previous year, such that

$$\overset{\wedge }{N}{}_{j,r,t+1}∼\text{Pois}\left(\lambda \right)$$

$$\lambda =\overset{\wedge }{N}{}_{a,r,t}\times R_{t}$$

$$R_{t}∼\text{Unif}\left(e^{-10},e^{10}\right)$$

where *R* was the recruitment rate. Because abundance estimates depended on estimates from the previous occasion, starting values (*t = 1*) for *N*_*j*_ and *N*_*a*_ were assumed to be equal to

$$N_{1}=\frac{H}{h}+1$$

### 2.2 Model validation

To assess model accuracy, we simulated data that spanned a series of adjacent regions with variable abundance. We generated a 100 km × 100 km virtual study area evenly divided into 25 regions (Figure S1). Capture sites were randomly distributed across the study area, and each was randomly assigned either a high, medium, or low number of captured individuals. For each data set, we simulated banding, telemetry, and total harvest data for a given population, and used constant intercepts and beta coefficients to simulate weekly survival and harvest rates across the area, with modifications as described below to incorporate spatial heterogeneity. To prevent unrealistic population growth, we restricted the maximum regional abundance using a fixed carrying capacity of 5000 individuals. To introduce spatial variation into harvest rate and survival parameters, we generated Gaussian random fields using the “gstat” package (Pebesma, 2004) in program R (R Core Team, 2020), which created a location‐specific beta coefficient that described spatial variation across the study area. We assessed the accuracy of estimates under variable sampling within a region and across the study area. To ensure that the simulation accurately presented a range of possible spatial heterogeneities and that the model was robust to those ranges of variation, we simulated multiple spatial configurations of harvest rate using low, medium, or high values for the partial sill, range, and nugget of the variogram used to generate the random field. In practice, this allowed us to vary the magnitude of variation in harvest rate, the maximum distance of autocorrelation, and the amount of small‐scale variation in harvest rates, respectively (Figure S2).

To evaluate model accuracy, we compared simulated values to estimates of harvest rate, survival, and abundance generated by the model. Due to the wide variation in potential abundance values among simulated regions, we did not use absolute measures of error, where regions with greater abundance would inherently have greater absolute error values. Instead, we calculated the relative bias as the difference between the true and estimated value for each region, divided by the true value.

$$\text{Relative Bias}=\frac{\text{True}-\text{Estimated}}{\text{True}}$$

To evaluate how the inclusion of an SPP to account for spatial heterogeneity in harvest rates impacted model estimates, we repeated the above analysis using a second model, which assumed a constant harvest rate across the study area (i.e., did not include an SPP). We then compared the relative bias between the two models.

### 2.3 Case study: Wild turkeys in Maine

To demonstrate the applicability of the model, we used data collected from wild turkeys in Maine, USA. Maine is a large state (~91,647 km^2^) with a variety of intermixed land use types and variable hunter densities. As such, we expected significant heterogeneity in harvest rates of turkeys within the state, as has been observed for other states' turkey populations (Stevens et al., 2020). Turkeys were captured during the winters (December through March) of 2018 through 2020 using rocket and drop nets and aged as either adult (>1 year old) or juvenile (<1 year old) according to plumage (Dickson, 1992). We marked turkeys with at least 1 of 4 different marking methods with associated identification numbers, including aluminum butt end leg bands, aluminum rivet bands, plastic colored leg bands, or patagial wing tags. Nearly all individuals received at least 2 marks, and we assume retention of at least 1 mark was 100%. In addition to identification numbers, leg bands included contact information (toll‐free phone number and web form) for hunters to report the harvest of banded individuals. Hunters could also report harvests when registering their turkey at state‐coordinated check stations.

At capture sites in the greater Portland and Bangor areas, a subset of turkeys was fit with 12 g VHF necklaces from Advanced Telemetry Systems (Model A3950; Isanti, Minnesota, USA), although 2 individuals were marked with 90 g Litetrack GPS backpack from Lotek Wireless Fish and Wildlife Monitoring (Newmarket, Ontario, CA). We attempted to record one live/dead status for each radioed individual per week. We censored individuals that died within the first 2 weeks after capture. Prior analysis of this population indicated that necklace transmitters do not affect male wild turkey survival postcapture (Gonnerman et al. *unpublished data*), and we assumed harvest rates were similar between turkeys fit with radio transmitters and those without.

In Maine, hunters were required to present their harvested turkeys to a local check station for registration, which provided both a count of total harvest within each of the state's Wildlife Management Districts (WMD), as well as age class designation within the total harvest. Total harvest information was available for all hunting seasons dating to 2006, from which we used data on total turkey harvest from 2011 through 2021. These years follow a series of changes to harvest regulations, which may impact estimates of harvest rate. Due to complications related to COVID‐19, MDIFW did not require hunters to register harvested turkeys during the 2020 spring hunting season. Instead, a survey was conducted to gauge success and estimate the total turkey harvest. We adjusted the model to treat 2020 total harvest data as a random variable with initial values equal to the estimated harvest from survey data. We do note that Maine also has a fall either‐sex hunting season, but harvest rates were generally much lower than spring, and we received an insufficient number of recoveries to build this into the model explicitly. Therefore survival reflects all mortality (nonharvest + fall harvest) occurring between the end of the spring hunting season in year *t* and the beginning of the subsequent spring season in *t + 1*.

In Maine, turkeys are managed within 29 discrete WMDs. For the distribution of spatial knots in the SPP, we used a grid with 24 km spacing, with additional knots placed at the geographic center of each WMD. We eliminated knots from WMDs, where turkey densities (<100) and harvests (<10) were expected to be insufficient to produce reliable estimates. These knot specifications ensured that each WMD of interest had at least one knot within its boundaries and that we did not predict harvest rates beyond where our data could reasonably be considered representative. The distance of 24 km was chosen by running multiple iterations of the model using various grid spacing. We then compared WMD‐specific harvest rate estimates for each iteration to a model with a categorical covariate for WMD. We used root mean squared error

$$\sum \sqrt{\frac{{\left(\mathrm{SPP}.\mathrm{est}–\text{Covariate}.\mathrm{est}\right)}^{2}}{n}}$$

to select the largest grid spacing that minimized error while also reaching convergence within the model. We compared estimates of harvest rate with and without the inclusion of the SPP component to assess whether failing to account for spatial variation in harvest rates affected parameter estimates.

### 2.4 Model fitting

We fit simulated data to the model, as described in section 2.1, using a Bayesian approach (Hobbs & Hooten, 2015). We used JAGS v.4.3.0 (Plummer, 2003) via the “R2jags” package (Su & Yajima, 2015) in the R v.4.0.3 programming environment (R Core Team, 2020). Regression coefficients were given vague uniform priors. Simulation models were allowed 10,000 iterations, discarding the first 5000 from calculations. The model fit to wild turkey data was allowed 50,000 iterations, discarding the first 20,000.

## 3 RESULTS

### 3.1 Simulation accuracy

**FIGURE 2:**
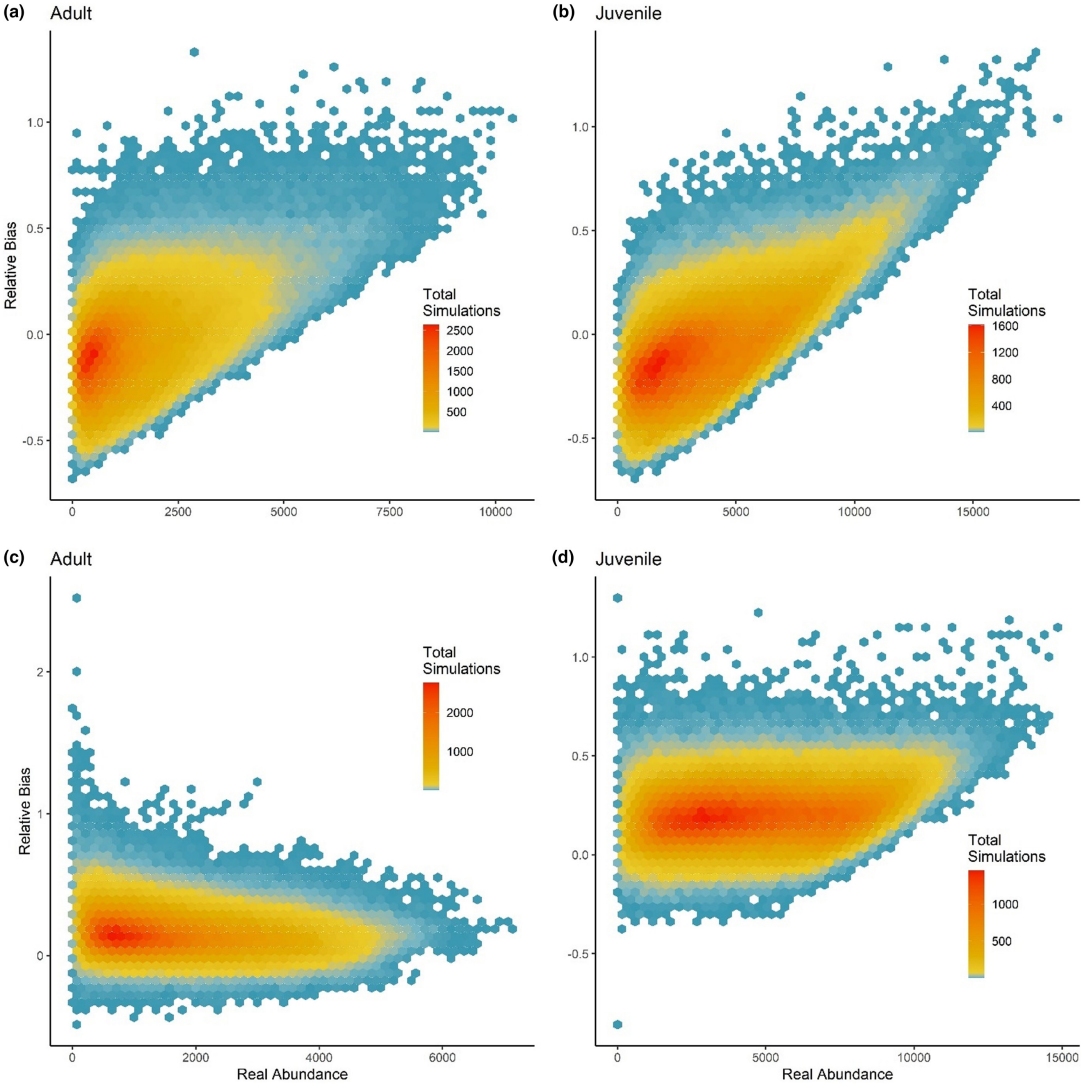
Relative bias for estimates of adult (a,c) and juvenile (b,d) abundance produced by a Lincoln estimator was on average less for a model with a spatial predictive process to account for spatial variation in harvest rates (a,b) compared with a model without (c,d). Number of simulations are depicted according to relative bias in region‐specific estimates of abundance and real abundance within the region. Results shown are grouped across simulated data sets, which varied in the underlying spatial heterogeneity in the harvest rate.

For all simulations that utilized the model with an SPP included, the average relative bias for abundance estimates was −0.04 (SD = 0.21) for adults and − 0.06 (SD = 0.22) for juveniles, with each being approximately zero‐centered (Figure 2a,b). Relative bias in harvest rate estimates averaged −0.10 (SD = 0.17) for adults and − 0.14 (SD = 0.22) for juveniles. Relative bias in weekly survival rate averaged 0.001 (SD = 0.002) for adults and 0.006 (SD = 0.005) for juveniles. We did not observe any relationship between relative bias in abundance estimates and configuration of spatial variation in harvest rates (Figure S3). Similarly, we did not observe any differences in relative bias in abundance associated with sampling intensity for the sample sizes we considered, both for sampling within a region and for sampling across a study area within a simulation (Figure S4). When we compared relative bias in abundance as it related to the portion of the population that was banded, we found that bias became more negative as the proportion banded increased and variance increased as the proportion banded decreased (Figure S5).

When the SPP was omitted from the model, the average relative bias for abundance estimates was 0.16 (SD = 0.14) for adults and 0.21 (SD = 0.13) for juveniles (Figure 2c,d, Figure S6). Relative bias in harvest rate estimates averaged 0.11 (SD = 0.09) for adults and 0.16 (SD = 0.16) for juveniles. Relative bias in weekly survival rate averaged −0.003 (SD = 0.003) for adults and 0.00 (SD = 0.004) for juveniles.

### 3.2 Case study

**FIGURE 3:**
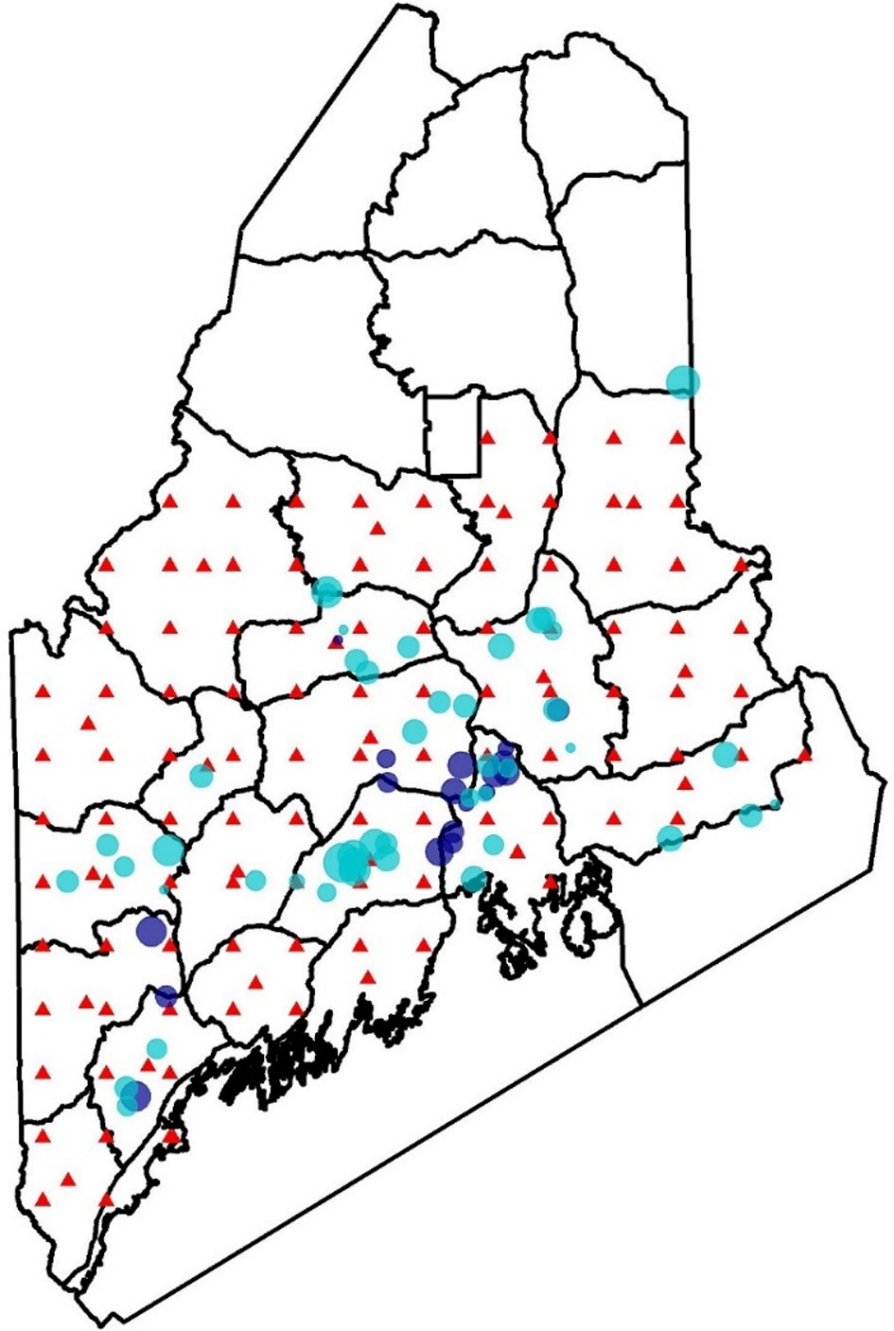
Spatial knots (red triangles) chosen for the spatial predictive process provide more uniform and complete coverage of the area of interest compared with capture sites (blue dots). Spatial knots were distributed across a 24 km × 24 km grid with additional knots placed at the geographic center of each WMD. We eliminated knots from WMDs where turkey densities (<100) and harvests (<10) were expected to be insufficient to produce reliable estimates. Sample size for capture sites is indicated by the size of the dot, with large dots meaning larger sample sizes. Color of dots indicates whether a site had telemetry devices deployed (dark blue) or did not (light blue).

We captured and marked 408 male wild turkeys (187 adults, 221 juveniles) at 72 capture sites across Maine (Figure 3). Transmitters were deployed on a subset of 58 males. We received a total of 136 reports of banded turkeys harvested during the 2018–2021 spring bearded turkey hunting seasons (33% of banded males), 12 of which were radio‐marked males (21% of radio‐marked males).

**FIGURE 4:**
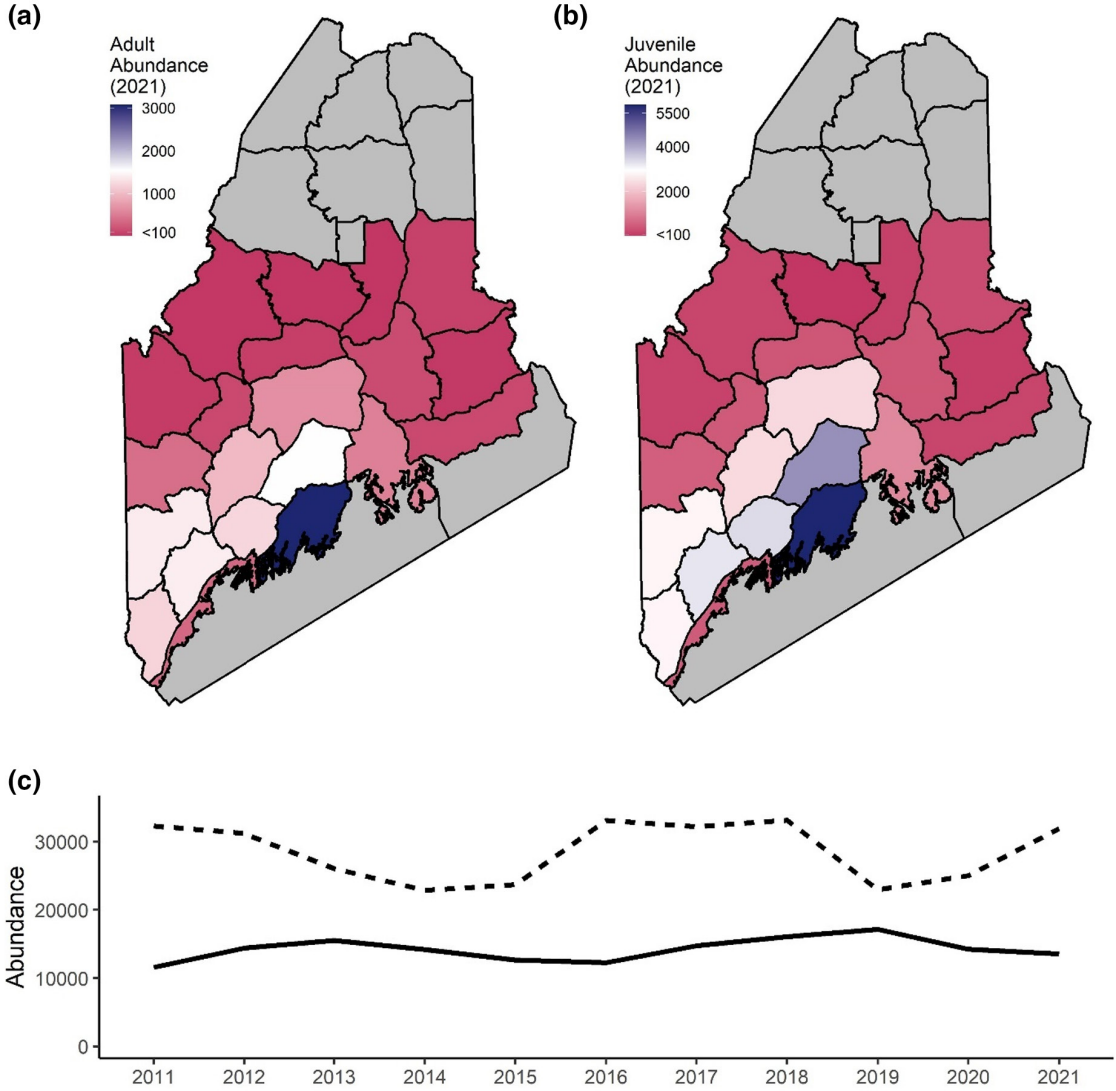
Wild turkey abundance varied across space and time for populations in Maine, USA, as predicted by a Lincoln estimator with a spatial predictive process component. Map colors indicate the mean abundance of wild turkey adults (a) and juveniles (b) in 2021. Adult (solid line) and juvenile (dashed line) turkey abundance is shown from 2011 through 2021 (c).

**FIGURE 5:**
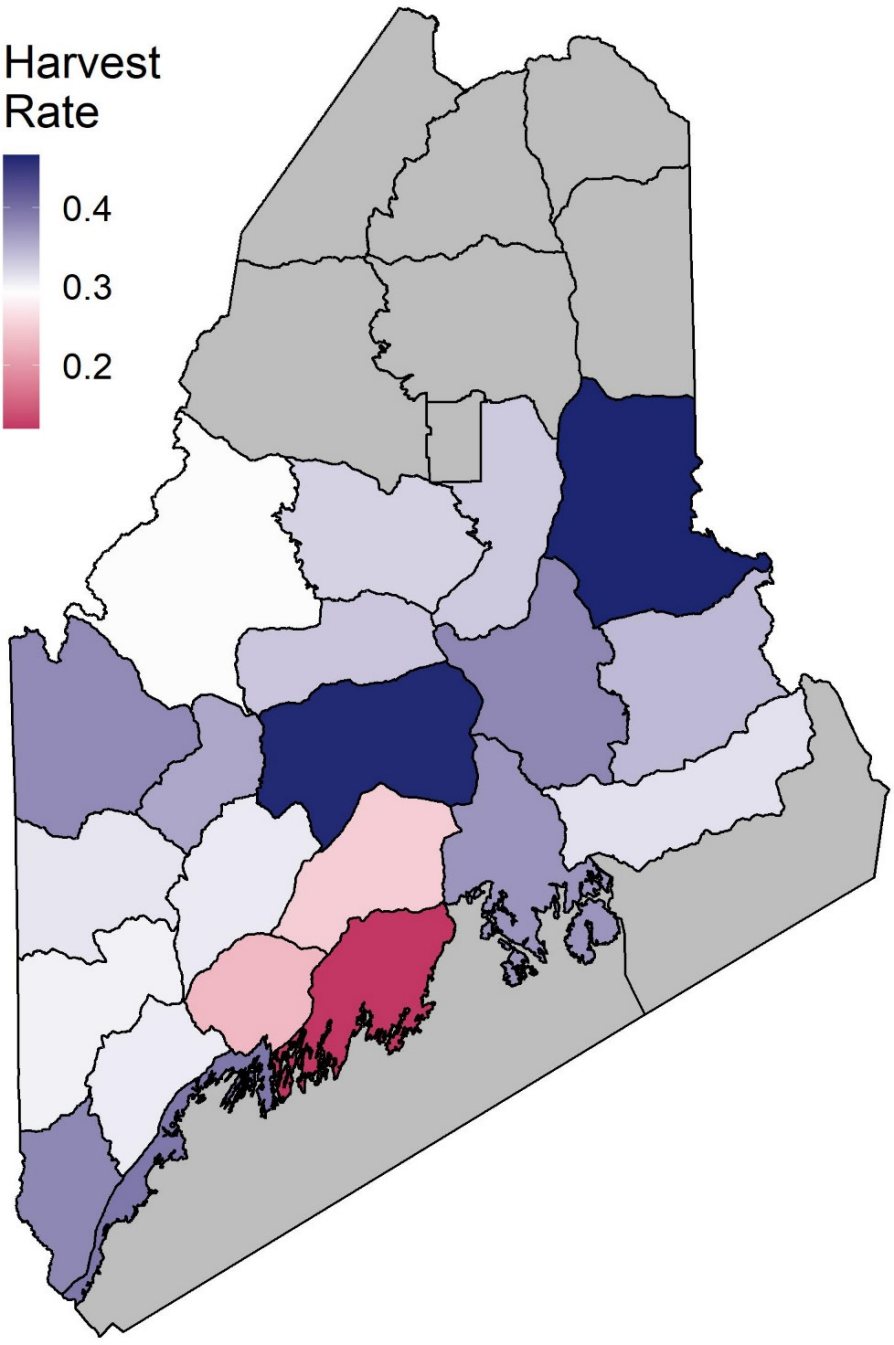
Harvest rate estimates were variable among wildlife management districts across Maine, USA, for both male and juvenile wild turkeys. Regional differences in harvest rates are depicted for adult male turkeys with color indicating the mean harvest rate for each management district.

Region‐specific estimates of turkey abundance averaged 677 adults and 1361 juveniles across all years and ranged from 2 to 4310 adults and 1 to 7010 juveniles (Figure 4). Statewide total male turkey abundance averaged 42,797 individuals and ranged between 36,338 turkeys in 2015 and 49,238 turkeys in 2018. Region‐specific estimates of adult harvest rates averaged 0.35 (ranged between 0.12–0.56; Figure 5) compared with 0.07 (ranged between 0.02 and 0.12) for juveniles. The mean weekly survival rate across years was 0.99 (ranged between 0.96 and 1.0) for adults and 0.98 (ranged between 0.89 and 1.0) for juveniles. Estimates of recruitment averaged 3.12 juveniles per adult and ranged between 0.12 and 18.18; this upper estimate, while clearly unrealistic, resulted from regions with extremely small population sizes. The 95th percentile of recruitment estimates ranged from 0.70 to 8.32.

**FIGURE 6:**
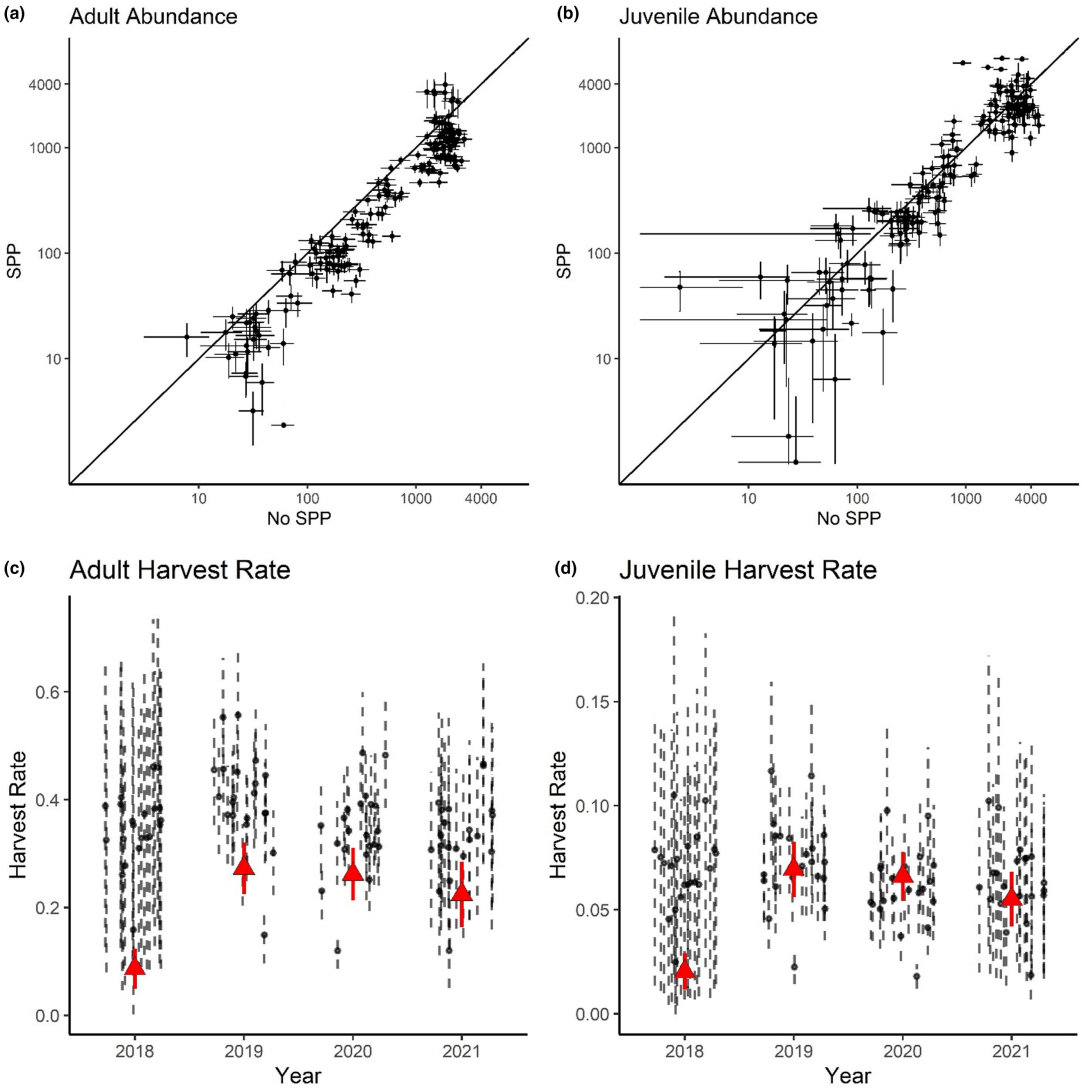
Estimates of adult abundance and harvest rate differed between Lincoln estimators with and without a spatial predictive process included, but juvenile harvest rate and abundance were largely similar. Estimates from both models are presented with associated error bars for abundance (a,b; shown on the log scale) and harvest rate (c,d). For all figures, circles correspond to WMD‐by‐year estimates. For c and d, triangles indicate the harvest rate estimated without the inclusion of the SPP, and circles indicate the SPP.

We found that the estimated abundance of adult turkeys was consistently lower and had narrower credible intervals, when the SPP was included in the model versus when it was not (Figure 6a). On average, the basic model without an SPP overestimated abundance by 286.96 (SD = 601.79) adults and 52.88 (SD = 1072.28) juveniles per WMD. Estimates of adult harvest rates were consistently higher when the model included an SPP compared with the model that assumed a constant harvest rate (Figure 6c). For juveniles, we observed substantially less difference in parameter estimates between models with and without an SPP (Figure 6b,d), consistent with a lower overall harvest rate for juvenile males with inherently less room for variability as a result.

## 4 DISCUSSION

We found that a Lincoln estimator incorporating an SPP in the band recovery model consistently estimated abundance with low bias across a variety of spatial configurations of harvest rate for simulated data. When the SPP was omitted from the model, relative bias in abundance increased for all configurations of spatial heterogeneity in harvest rate. Additionally, we found no difference in relative bias according to either sampling intensity or the underlying nature of spatial heterogeneity in harvest rate. When applied to real data collected from turkeys in Maine, we observed a wide range of harvest rates and abundance among wildlife management districts. As expected, the basic model that did not incorporate spatial heterogeneity in harvest rates via the SPP underestimated harvest rates in some WMDs, which resulted in an overestimation of abundance in those districts, and statewide. The variation in harvest rates we observed is typical for wild turkey populations and should be expected especially across large spatial scales (Norton et al., 2012). Most recent applications of Lincoln estimators have treated harvest rates as uniform across large areas (Alisauskas et al., 2014; Hagen et al., 2018; Shirkey & Gates, 2020). While this assumption may sometimes be appropriate and will likely depend on the definition of “large areas,” our results suggest that future applications could consider more explicitly incorporating spatial variation in harvest rates to improve inference. While we chose to use an SPP to accomplish this, other methods such as simultaneous autoregressive and conditional autoregressive models may also be a viable option (Fortin et al., 2012).

Harvest management decisions must often consider populations that span considerably large spatial scales (Robinson et al., 2016), and it may not be necessary to reconcile inference at the local scale when decisions are made at regional levels (Johnson et al., 2015). Instead, estimates can be aggregated to summarize relationships within a region's boundaries, making fine‐scale differences in parameters at local scales less important than adequately capturing the general trend in a parameter across space. Aggregating estimates to describe parameters by region can be performed using multiple methods, with the simplest solution being to average estimates within each region. However, consideration must be given to the sampling design used, as clumped or sparse sampling within an area could lead to bias if sampled locations differ greatly from the mean across a landscape (Hooten et al., 2017). To some degree, SPP can mitigate such issues by using the entire data set to define a spatial correlation function and projecting it onto evenly distributed spatial knots from which estimates are then made. This will have the benefit of smoothing the prediction surface, minimizing the impact of any single sampling location, which may otherwise have outsized impacts on local averages. However, if larger areas or districts have particularly high or low harvest rates, and are unsampled, the model would not be able to interpolate those relationships, meaning that adequate and representative sampling is still an important component of study design. When adequate sampling is performed, SPP has proven to be effective at identifying localized “hotspots” in variation (Viana et al., 2013), and indeed we found that model predictions were robust to a wide range of underlying spatial heterogeneity in harvest rate.

We observed some variation in the magnitude and direction of error across iterations, which is common when assessing IPMs (Abadi et al., 2010; Fieberg et al., 2010). We further found that relative bias became more negative as the proportion of a population that was banded increased. This shift in bias is consistent with a long‐understood relationship, where the accuracy of mark‐recapture models depends on the ratio of the banded sample to total abundance (Robson & Regier, 1964), and reinforces previous recommendations that sample size of banding studies should be informed by expected population size (Robson & Regier, 1964).

Despite the advantages of SPP, there are still opportunities for improvement. Although SPP should be notably faster than alternatives, especially as the amount of data increases, using MCMC can still lead to lengthy computing times (Banerjee et al., 2008) so alternative posterior samplers should be considered to decrease processing time. The advantages of SPP will not overcome extremely sparse data availability or poor sampling design. As previously mentioned, the number of individuals marked should be proportional to the expected population size being sampled. While we did not observe an effect of sample size on the model bias for the sample sizes we considered, further exploration with more limited data sets may be necessary to find a threshold at which estimates are no longer reliable. Similarly, the distribution of sampling locations and the configuration of spatial knots across a study area should be informed by the ecology of the system being studied. The number and placement of knots for the SPP are not trivial, and while there appears to be a wide margin for error, these decisions have an impact on estimates (Banerjee et al., 2008). For example, we chose to use the capture sites as input locations for the SPP as we did not have accurate information on locations where harvests occurred. For turkeys that move relatively short distances, this is unlikely to be an issue, but migratory species may require more detailed information on harvest locations as the distances between capture and harvest may be much greater. We also note that in situations where multiple parameters influence an outcome, such as harvest and survival in a band recovery model, failure to account for experiencing spatial heterogeneity in one parameter could result in increased variation in estimates of the other.

Multiple methods are currently used to monitor turkey populations. Many states approximate population trends using spring harvest data (e.g., CDEEP, 2016; Harms et al., 2017; Healy, 2000), but this does not provide an estimate of true abundance, which may be preferable for setting regulations (Lint et al., 1995). This method also requires accounting for changes in the abundance of birds and harvest rates, both of which influence the number of birds harvested through time (Paloheimo & Fraser, 1981). Surveys such as summer sighting (PGC, 2021), gobble counts (Rioux et al., 2009), and camera traps (Gonnerman, 2017) can be used to produce estimates of population size at smaller scales but are unrealistic to implement for statewide management. An IPM, such as the one we have implemented, provides a data‐driven alternative that can be scaled to the scope of turkey management decisions. It is relatively cost‐effective as it uses often already implemented mandatory reporting of harvests and only requires periodic captures of individuals for banding. Similar IPMs have been implemented for turkey and waterfowl populations to great success (Arnold et al., 2018; Diefenbach et al., 2012), demonstrating that this is a feasible alternative that, with the inclusion of an SPP component, overcomes many of the shortcomings of more common monitoring methods. We imagine this approach may have more broad applicability to other similarly‐managed harvest systems, such as those for large mammals, where regional or subregional variation in harvest regulations is common.

## 5 CONCLUSIONS

Management decisions based on biased estimates of abundance may lead to harvest regulations that exceed sustainable levels or are unnecessarily restrictive (Dillingham & Fletcher, 2008). Violations of the Lincoln estimator's assumption of representative harvest may result in such bias. As Lincoln estimators become more widely applied, it is important to consider a mechanism to account for spatial variation in harvest rates, which can vary according to a broad range of spatially varying ecological, environmental, and socio‐economic factors that are sometimes difficult to measure (Pope & Powell, 2021). For such cases, the combination of Lincoln estimator and SPP is an especially relevant tool for capturing the magnitude and distribution of variation to reduce bias in estimates used for management. SPP functions as a component of a generalized linear mixed model framework, making it compatible with many analytical methods currently used in ecology, and therefore should be accessible to those who are less familiar with spatial statistics. While we chose to apply these methods to harvest rates within a band recovery model, the use of SPP should be widely applicable across methods for vital rate estimation.

## AUTHOR CONTRIBUTIONS

**Matthew Gonnerman:** Conceptualization (equal); data curation (equal); formal analysis (equal); methodology (equal); visualization (lead); writing – original draft (lead); writing – review and editing (equal). **Daniel W Linden:** Formal analysis (equal); methodology (equal); writing – original draft (equal). **Stephanie Shea:** Data curation (equal); project administration (equal); writing – review and editing (equal). **Kelsey Sullivan:** Conceptualization (equal); data curation (equal); funding acquisition (equal); investigation (equal); methodology (equal); project administration (equal); supervision (equal); writing – review and editing (equal). **Pauline Kamath:** Data curation (equal); project administration (equal); supervision (equal); writing – review and editing (equal). **Erik Blomberg:** Conceptualization (equal); data curation (equal); formal analysis (equal); funding acquisition (equal); investigation (equal); project administration (equal); supervision (equal); writing – original draft (equal); writing – review and editing (lead).

## CONFLICT OF INTEREST

All authors involved attest to having no conflicts of interest related to the submitted manuscript.

## Supporting information


Figure S1–S6

## Data Availability

The data that support the findings of this study are openly available in GitHub at https://github.com/mattgonnerman/BandRecoveryModel (Gonnerman, 2022).

## References

[ece39444-bib-0001] Abadi, F. , Gimenez, O. , Arlettaz, R. , & Schaub, M. (2010). An assessment of integrated population models: Bias, accuracy, and violation of the assumption of independence. Ecology, 91(1), 7–14. 10.1890/08-2235.1 20380189

[ece39444-bib-0002] Alisauskas, R. T. , Arnold, T. W. , Leafloor, J. O. , Otis, D. L. , & Sedinger, J. S. (2014). Lincoln estimates of mallard (Anas platyrhynchos) abundance in North America. Ecology and Evolution, 4(2), 132–143. 10.1002/ece3.906 24558569 PMC3925377

[ece39444-bib-0003] Alisauskas, R. T. , Drake, K. L. , & Nichols, J. D. (2009). Filling a void: Abundance estimation of north American populations of Arctic geese using hunter recoveries. In Modeling demographic processes in marked populations (Vol. 3, pp. 463–489). Springer.

[ece39444-bib-0004] Arnold, T. W. , Clark, R. G. , Koons, D. N. , & Schaub, M. (2018). Integrated population models facilitate ecological understanding and improved management decisions. The Journal of Wildlife Management, 82(2), 266–274. 10.1002/jwmg.21404

[ece39444-bib-0005] Banerjee, S. , Gelfand, A. E. , Finley, A. O. , & Sang, H. (2008). Gaussian predictive process models for large spatial data sets. Journal of the Royal Statistical Society: Series B (Statistical Methodology), 70(4), 825–848. 10.1111/j.1467-9868.2008.00663.x 19750209 PMC2741335

[ece39444-bib-0006] Bled, F. , Belant, J. L. , Daele, L. J. V. , Svoboda, N. , Gustine, D. , Hilderbrand, G. , & Barnes, V. G. (2017). Using multiple data types and integrated population models to improve our knowledge of apex predator population dynamics. Ecology and Evolution, 7(22), 9531–9543. 10.1002/ece3.3469 29187987 PMC5696435

[ece39444-bib-0007] Brownie, C. (1985). Statistical inference from band recovery data: A handbook. Resource Publication/United States Department of the Interior. https://agris.fao.org/agris‐search/search.do?recordID=US9017008

[ece39444-bib-0008] Buderman, F. E. , Diefenbach, D. R. , Casalena, M. J. , Rosenberry, C. S. , & Wallingford, B. D. (2014). Accounting for tagging‐to‐harvest mortality in a Brownie tag‐recovery model by incorporating radio‐telemetry data. Ecology and Evolution, 4(8), 1439–1450. 10.1002/ece3.1025 24834339 PMC4020702

[ece39444-bib-0009] Burke, C. R. , Peterson, M. N. , Sawyer, D. T. , Moorman, C. E. , Serenari, C. , & Pacifici, K. (2019). A method for mapping hunting occurrence using publicly available, geographic variables. Wildlife Society Bulletin, 43(3), 537–545. 10.1002/wsb.994

[ece39444-bib-0010] Burrough, P. A. (1995). Spatial aspects of ecological data. In C. J. F. T. Braak , O. F. R. van Tongeren , & R. H. G. Jongman (Eds.), Data analysis in community and landscape ecology (pp. 213–251). Cambridge University Press. 10.1017/CBO9780511525575.009

[ece39444-bib-0011] Chandler, R. B. , & Clark, J. D. (2014). Spatially explicit integrated population models. Methods in Ecology and Evolution, 5(12), 1351–1360. 10.1111/2041-210X.12153

[ece39444-bib-0012] Connecticut Department of Energy and Environmental Protection [CDEEP] . (2016). Connecticut wild Turkey program report: 2016 Spring and fall seasons. https://portal.ct.gov/‐/media/DEEP/wildlife/pdf_files/game/turksum2016pdf.pdf

[ece39444-bib-0013] Cooch, E. G. , Alisauskas, R. T. , & Buderman, F. E. (2021). Effect of pre‐harvest mortality on harvest rates and derived population estimates. The Journal of Wildlife Management, 85(2), 228–239. 10.1002/jwmg.21986

[ece39444-bib-0014] Cressie, N. (2015). Statistics for spatial data revised edition. John Wiley & Sons.

[ece39444-bib-0015] Dickson, J. G. (1992). The wild Turkey: Biology and management. Stackpole Books.

[ece39444-bib-0016] Diefenbach, D. R. , Casalena, M. J. , Schiavone, M. V. , Reynolds, M. , Eriksen, R. , Vreeland, W. C. , Swift, B. , & Boyd, R. C. (2012). Variation in spring harvest rates of male wild turkeys in New York, Ohio, and Pennsylvania. The Journal of Wildlife Management, 76(3), 514–522. 10.1002/jwmg.256

[ece39444-bib-0017] Dillingham, P. W. , & Fletcher, D. (2008). Estimating the ability of birds to sustain additional human‐caused mortalities using a simple decision rule and allometric relationships. Biological Conservation, 141(7), 1783–1792. 10.1016/j.biocon.2008.04.022

[ece39444-bib-0018] Dinsmore, S. J. , White, G. C. , & Knopf, F. L. (2002). Advanced techniques for modeling avian Nest survival. Ecology, 83(12), 3476–3488. 10.1890/0012-9658(2002)083[3476:ATFMAN]2.0.CO;2

[ece39444-bib-0019] Ergon, T. , Borgan, Ø. , Nater, C. R. , & Vindenes, Y. (2018). The utility of mortality hazard rates in population analyses. Methods in Ecology and Evolution, 9(10), 2046–2056. 10.1111/2041-210X.13059

[ece39444-bib-0020] Fay, R. , Michler, S. , Laesser, J. , & Schaub, M. (2019). Integrated population model reveals that kestrels breeding in nest boxes operate as a source population. Ecography, 42(12), 2122–2131. 10.1111/ecog.04559

[ece39444-bib-0021] Fieberg, J. R. , Shertzer, K. W. , Conn, P. B. , Noyce, K. V. , & Garshelis, D. L. (2010). Integrated population modeling of black bears in Minnesota: Implications for monitoring and management. PLoS One, 5(8), e12114. 10.1371/journal.pone.0012114 20711344 PMC2920827

[ece39444-bib-0022] Finley, A. O. , Sang, H. , Banerjee, S. , & Gelfand, A. E. (2009). Improving the performance of predictive process modeling for large datasets. Computational Statistics & Data Analysis, 53(8), 2873–2884. 10.1016/j.csda.2008.09.008 20016667 PMC2743161

[ece39444-bib-0023] Fleskes, J. P. , Yee, J. L. , Yarris, G. S. , Miller, M. R. , & Casazza, M. L. (2007). Pintail and mallard survival in California relative to habitat, abundance, and hunting. The Journal of Wildlife Management, 71(7), 2238–2248. 10.2193/2005-634

[ece39444-bib-0024] Fortin, M.‐J. , James, P. M. A. , MacKenzie, A. , Melles, S. J. , & Rayfield, B. (2012). Spatial statistics, spatial regression, and graph theory in ecology. Spatial Statistics, 1, 100–109. 10.1016/j.spasta.2012.02.004

[ece39444-bib-0025] Freeman, S. N. , & Crick, H. Q. P. (2003). The decline of the spotted flycatcher Muscicapa striata in the UK: An integrated population model. Ibis, 145(3), 400–412. 10.1046/j.1474-919X.2003.00177.x

[ece39444-bib-0026] Gonnerman, M. (2017). Estimating use, density, and productivity of eastern wild Turkey in Alabama. https://etd.auburn.edu//handle/10415/5942

[ece39444-bib-0027] Gonnerman, M. (2022). Including a spatial predictive process in band recovery models improves inference for Lincoln estimates of animal abundance, Dryad, Dataset. 10.5061/dryad.9p8cz8wkp PMC9608798 36311403

[ece39444-bib-0028] Hagen, C. , Sedinger, J. , & Braun, C. (2018). Estimating sex‐ratio, survival, and harvest susceptibility in greater sage‐grouse: Making the most of hunter harvests. Wildlife Biology, 2018(1), 1–7. 10.2981/wlb.00362.full

[ece39444-bib-0029] Hagen, R. , Kramer‐Schadt, S. , Fahse, L. , & Heurich, M. (2014). Population control based on abundance estimates: Frequency does not compensate for uncertainty. Ecological Complexity, 20, 43–50. 10.1016/j.ecocom.2014.07.006

[ece39444-bib-0030] Hansen, L. P. , Nixon, C. M. , & Loomis, F. (1986). Factors affecting daily and annual harvest of White‐tailed deer in Illinois. Wildlife Society Bulletin, 14(4), 368–376. https://www.jstor.org/stable/3782268

[ece39444-bib-0031] Harms, T. , Coffey, J. , Evelsizer, V. , Jones, O. , Bogenschutz, T. , Shepherd, S. , Schlarbaum, P. , Ehresman, B. , Shepherd, S. , Ehresman, B. , Evelsizer, V. , Evelsizer, V. , Evelsizer, V. , Hoffman, D. , & Norton, A. (2017). Trends in Iowa wildlife populations and harvest 2016–2017. Iowa Department of Natural Resources. https://www.iowadnr.gov/Portals/idnr/uploads/Hunting/trends/logbook_2016.pdf

[ece39444-bib-0032] Healy, W. M. (2000). Wild Turkey harvest management: Biology, strategies, and techniques. Fish & Wildlife Service.

[ece39444-bib-0033] Hobbs, N. T. , & Hooten, M. B. (2015). Bayesian models: A statistical primer for ecologists. Princeton University Press.

[ece39444-bib-0034] Hooten, M. B. , Johnson, D. S. , McClintock, B. T. , & Morales, J. M. (2017). Animal movement: Statistical models for telemetry data. CRC Press. 10.1201/9781315117744

[ece39444-bib-0035] Horne, J. S. , Ausband, D. E. , Hurley, M. A. , Struthers, J. , Berg, J. E. , & Groth, K. (2019). Integrated population model to improve knowledge and management of Idaho wolves. The Journal of Wildlife Management, 83(1), 32–42. 10.1002/jwmg.21554

[ece39444-bib-0036] Hostetler, J. A. , & Chandler, R. B. (2015). Improved state‐space models for inference about spatial and temporal variation in abundance from count data. Ecology, 96(6), 1713–1723. 10.1890/14-1487.1

[ece39444-bib-0037] Humberg, L. A. , Devault, T. L. , & Jr, O. E. R. (2009). Survival and cause‐specific mortality of wild turkeys in northern Indiana. The American Midland Naturalist, 161(2), 313–322. 10.1674/0003-0031-161.2.313

[ece39444-bib-0038] Jarzyna, M. A. , Finley, A. O. , Porter, W. F. , Maurer, B. A. , Beier, C. M. , & Zuckerberg, B. (2014). Accounting for the space‐varying nature of the relationships between temporal community turnover and the environment. Ecography, 37(11), 1073–1083. 10.1111/ecog.00747

[ece39444-bib-0039] Johnson, F. A. , Boomer, G. S. , Williams, B. K. , Nichols, J. D. , & Case, D. J. (2015). Multilevel learning in the adaptive Management of Waterfowl Harvests: 20 years and counting. Wildlife Society Bulletin, 39(1), 9–19. 10.1002/wsb.518

[ece39444-bib-0040] Johnson, F. A. , Walters, M. A. H. , & Boomer, G. S. (2012). Allowable levels of take for the trade in Nearctic songbirds. Ecological Applications, 22(4), 1114–1130. 10.1890/11-1164.1 22827122

[ece39444-bib-0041] Latimer, A. M. , Banerjee, S. , Sang, H., Jr. , Mosher, E. S. , & Silander, J. A., Jr. (2009). Hierarchical models facilitate spatial analysis of large data sets: A case study on invasive plant species in the northeastern United States. Ecology Letters, 12(2), 144–154. 10.1111/j.1461-0248.2008.01270.x 19143826

[ece39444-bib-0042] Lee, A. M. , Bjørkvoll, E. M. , Hansen, B. B. , Albon, S. D. , Stien, A. , Sæther, B. E. , Engen, S. , Veiberg, V. , Loe, L. E. , & Grøtan, V. (2015). An integrated population model for a long‐lived ungulate: More efficient data use with Bayesian methods. Oikos, 124(6), 806–816. 10.1111/oik.01924

[ece39444-bib-0043] Lincoln, F. C. (1930). Calculating waterfowl abundance on the basis of banding returns (vol. 118). US Department of Agriculture. http://www.jstor.org/stable/4155929

[ece39444-bib-0044] Lint, J. R. , Leopold, B. D. , & Hurst, G. A. (1995). Comparison of abundance indexes and population estimates for wild Turkey gobblers. Wildlife Society Bulletin (1973–2006), 23(2), 164–168. http://www.jstor.org/stable/3782783

[ece39444-bib-0045] Nichols, J. D. , Runge, M. C. , Johnson, F. A. , & Williams, B. K. (2007). Adaptive harvest management of north American waterfowl populations: A brief history and future prospects. Journal of Ornithology, 148(2), 343–349. 10.1007/s10336-007-0256-8

[ece39444-bib-0046] Norton, A. S. , Diefenbach, D. R. , Wallingford, B. D. , & Rosenberry, C. S. (2012). Spatio‐temporal variation in male white‐tailed deer harvest rates in Pennsylvania: Implications for estimating abundance. The Journal of Wildlife Management, 76(1), 136–143. 10.1002/jwmg.249

[ece39444-bib-0047] Otis, D. L. (2006). Mourning dove hunting regulation strategy based on annual harvest statistics and banding data. Journal of Wildlife Management, 70(5), 1302–1307. 10.2193/0022-541X(2006)70[1302:MDHRSB]2.0.CO;2

[ece39444-bib-0048] Paloheimo, J. E. , & Fraser, D. (1981). Estimation of harvest rate and vulnerability from age and sex data. The Journal of Wildlife Management, 45(4), 948–958. 10.2307/3808102

[ece39444-bib-0049] Pebesma, E. J. (2004). Multivariable geostatistics in S: The gstat package. Computers & Geosciences, 30(7), 683–691. 10.1016/j.cageo.2004.03.012

[ece39444-bib-0050] Pennsylvania Game Commission [PGC] . (2021). Pennsylvania wild Turkey. Pennsylvania Game Commission. https://www.pgc.pa.gov/Wildlife/WildlifeSpecies/Turkey/Pages/default.aspx

[ece39444-bib-0051] Perkins, A. L. , Clark, W. R. , Riley, T. Z. , & Vohs, P. A. (1997). Effects of landscape and weather on winter survival of ring‐necked pheasant hens. The Journal of Wildlife Management, 61(3), 634–644. 10.2307/3802171

[ece39444-bib-0052] Plummer, M. (2003). JAGS: A program for analysis of Bayesian graphical models using Gibbs sampling. Proceedings of the 3rd international workshop on distributed statistical computing, 124(125.10), 1–10.

[ece39444-bib-0053] Pollock, K. H. , & Raveling, D. G. (1982). Assumptions of modern band‐recovery models, with emphasis on heterogeneous survival rates. The Journal of Wildlife Management, 46(1), 88–98. 10.2307/3808411

[ece39444-bib-0054] Pope, K. L. , & Powell, L. A. (2021). Harvest of fish and wildlife: New paradigms for sustainable management. CRC Press.

[ece39444-bib-0055] R Core Team . (2020). R: A language and environment for statistical computing (4.0.3). R Foundation for Statistical Computing. https://www.R‐project.org/

[ece39444-bib-0056] Riecke, T. V. , Williams, P. J. , Behnke, T. L. , Gibson, D. , Leach, A. G. , Sedinger, B. S. , Street, P. A. , & Sedinger, J. S. (2019). Integrated population models: Model assumptions and inference. Methods in Ecology and Evolution, 10(7), 1072–1082. 10.1111/2041-210X.13195

[ece39444-bib-0057] Rioux, S. , Bélisle, M. , & Giroux, J.‐F. (2009). Effects of landscape structure on male density and spacing patterns in wild turkeys (Meleagris gallopavo) depend on winter severity. The Auk, 126(3), 673–683. 10.1525/auk.2009.08127

[ece39444-bib-0058] Roberts, A. J. , Dooley, J. L. , Ross, B. E. , Nichols, T. C. , Leafloor, J. O. , & Dufour, K. W. (2021). An integrated population model for harvest Management of Atlantic Brant. The Journal of Wildlife Management, 85(5), 897–908. 10.1002/jwmg.22037

[ece39444-bib-0059] Robinson, K. F. , Fuller, A. K. , Hurst, J. E. , Swift, B. L. , Kirsch, A. , Farquhar, J. , Decker, D. J. , & Siemer, W. F. (2016). Structured decision making as a framework for large‐scale wildlife harvest management decisions. Ecosphere, 7(12), e01613. 10.1002/ecs2.1613

[ece39444-bib-0060] Robson, D. S. , & Regier, H. A. (1964). Sample size in Petersen mark–recapture experiments. Transactions of the American Fisheries Society, 93(3), 215–226. 10.1577/1548-8659(1964)93[215:SSIPME]2.0.CO;2

[ece39444-bib-0061] Runge, M. C. , Sauer, J. R. , Avery, M. L. , Blackwell, B. F. , & Koneff, M. D. (2009). Assessing allowable take of migratory birds. The Journal of Wildlife Management, 73(4), 556–565. 10.2193/2008-090

[ece39444-bib-0062] Saunders, S. P. , Farr, M. T. , Wright, A. D. , Bahlai, C. A. , Ribeiro, J. W. , Rossman, S. , Sussman, A. L. , Arnold, T. W. , & Zipkin, E. F. (2019). Disentangling data discrepancies with integrated population models. Ecology, 100(6), e02714. 10.1002/ecy.2714 30927256

[ece39444-bib-0063] Schaub, M. , & Abadi, F. (2011). Integrated population models: A novel analysis framework for deeper insights into population dynamics. Journal of Ornithology, 152(1), 227–237. 10.1007/s10336-010-0632-7

[ece39444-bib-0064] Shirkey, B. T. , & Gates, R. J. (2020). Survival, harvest, and Lincoln estimates of wood ducks banded in Ohio. Journal of Fish & Wildlife Management, 11(1), 185–195. 10.3996/082019-JFWM-070

[ece39444-bib-0065] Stevens, B. S. , Luukkonen, D. R. , Stewart, C. A. , Porter, W. F. , Bence, J. R. , & Jones, M. L. (2020). Spatial–temporal dynamics of hunter effort for wild turkeys in Michigan. PLoS One, 15(4), e0230747. 10.1371/journal.pone.0230747 32236108 PMC7112203

[ece39444-bib-0066] Su, Y.‐S. , & Yajima, M. (2015). R2jags: Using R to run ‘JAGS’. R Package Version, 0(5–7), 34.

[ece39444-bib-0067] Tavecchia, G. , Besbeas, P. , Coulson, T. , Morgan, B. J. T. , & Clutton‐Brock, T. H. (2009). Estimating population size and hidden demographic parameters with state‐space modeling. The American Naturalist, 173(6), 722–733. 10.1086/598499 19355815

[ece39444-bib-0068] Thogmartin, W. E. , Howe, F. P. , James, F. C. , Johnson, D. H. , Reed, E. T. , Sauer, J. R. , & Thompson III, F. R. (2006). A review of the population estimation approach of the north American Landbird conservation plan. The Auk, 123(3), 892–904. 10.1093/auk/123.3.892

[ece39444-bib-0069] Tolon, V. T. , Dray, S. D. , Loison, A. L. , Zeileis, A. Z. , Fischer, C. F. , & Baubet, E. B. (2009). Responding to spatial and temporal variations in predation risk: Space use of a game species in a changing landscape of fear. Canadian Journal of Zoology, 87, 1129–1137. 10.1139/Z09-101

[ece39444-bib-0070] Viana, M. , Jackson, A. L. , Graham, N. , & Parnell, A. C. (2013). Disentangling spatio‐temporal processes in a hierarchical system: A case study in fisheries discards. Ecography, 36, 569–578. 10.1111/j.1600-0587.2012.07853.x

[ece39444-bib-0071] Weinbaum, K. Z. , Brashares, J. S. , Golden, C. D. , & Getz, W. M. (2013). Searching for sustainability: Are assessments of wildlife harvests behind the times? Ecology Letters, 16(1), 99–111. 10.1111/ele.12008 23062121 PMC3521087

[ece39444-bib-0072] Wilson, S. , Gil‐Weir, K. C. , Clark, R. G. , Robertson, G. J. , & Bidwell, M. T. (2016). Integrated population modeling to assess demographic variation and contributions to population growth for endangered whooping cranes. Biological Conservation, 197, 1–7. 10.1016/j.biocon.2016.02.022

[ece39444-bib-0073] Zipkin, E. F. , & Saunders, S. P. (2018). Synthesizing multiple data types for biological conservation using integrated population models. Biological Conservation, 217, 240–250. 10.1016/j.biocon.2017.10.017

