# Supplementary material for: Including a spatial predictive process in band recovery models improves inference for Lincoln estimates of animal abundance
Source: Ecol Evol. 2022 Oct 27;12(10):e9444. doi: 10.1002/ece3.9444 (PMC9608798; doi:10.1002/ece3.9444)
Supplement: Supplementary file 1 — Figure S1–S6 [file ECE3-12-e9444-s001.docx]

APPENDIX


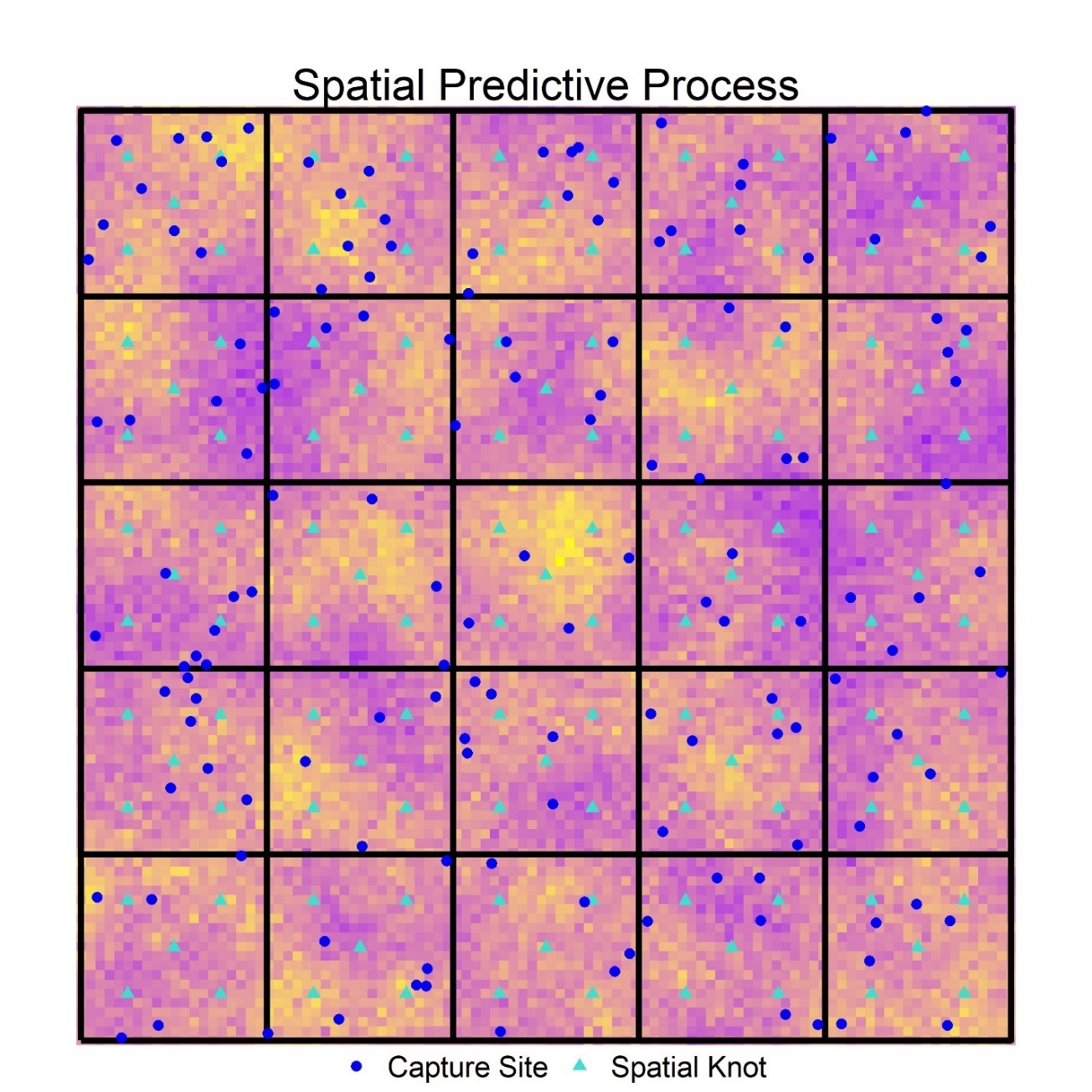


Figure S1. Example spatial predictive process from simulation approach depicting the projection of the underlying spatial correlation between capture sites (dots) onto an evenly distributed number of spatial knots (triangles) which generates parameter estimates that are more representative of the area as a whole. Simulated capture sites are distributed randomly across equally sized regions to reduce impact of survey design on estimates. Raster colors correspond to varying values for harvest rate, where darker colors indicate greater values for harvest rate.


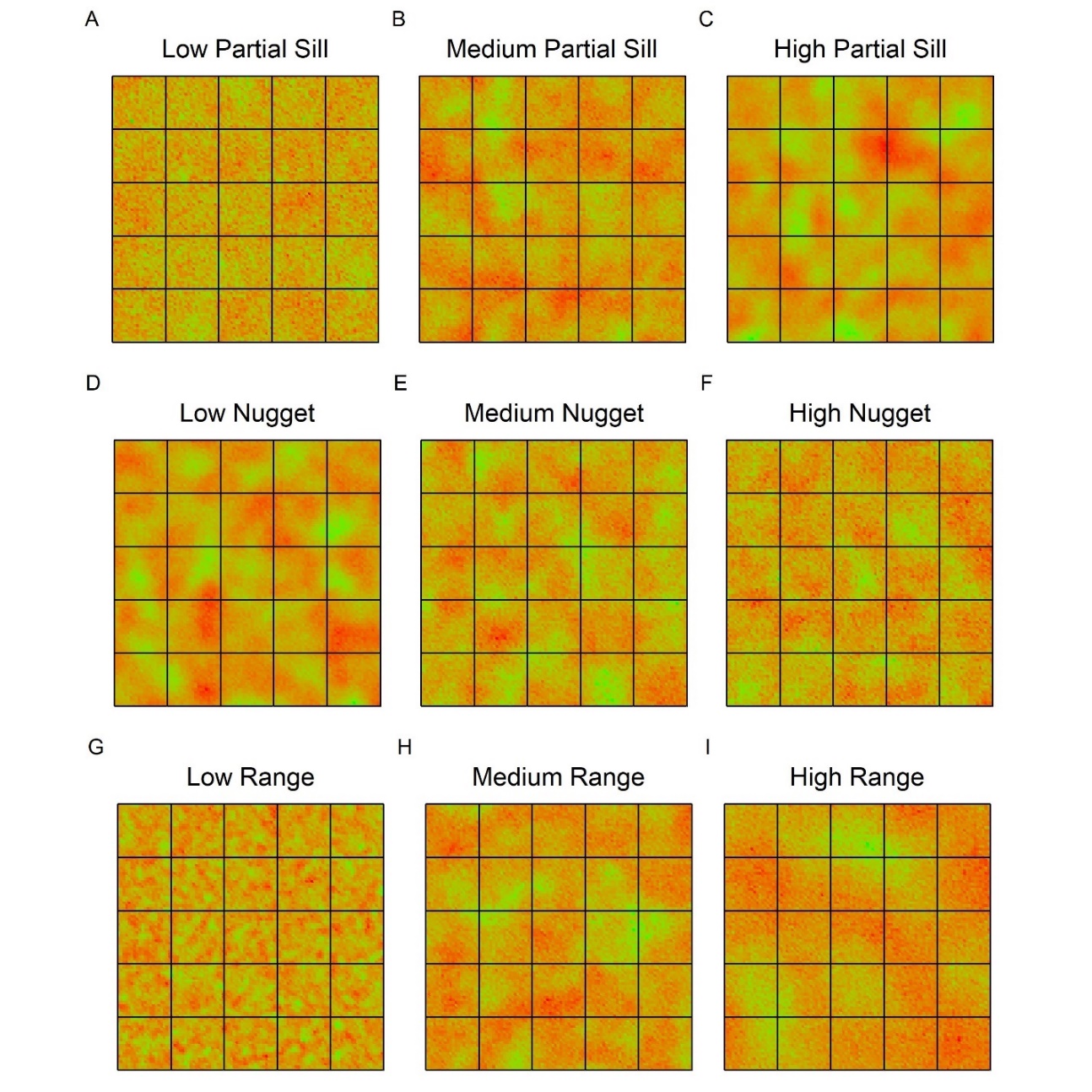


Figure S2. Spatial variation can follow many configurations, as shown by these example maps depicting potential variation in a parameter of interest described as a random gaussian process. Differences in values across the surface relate to aspects of the variogram describing the gausian process which controls differences in the magnitude of variation (partial sill; A-C), the maximum distance of autocorrelation (nugget; D-F), and the amount of small-scale variation (range; G-I).


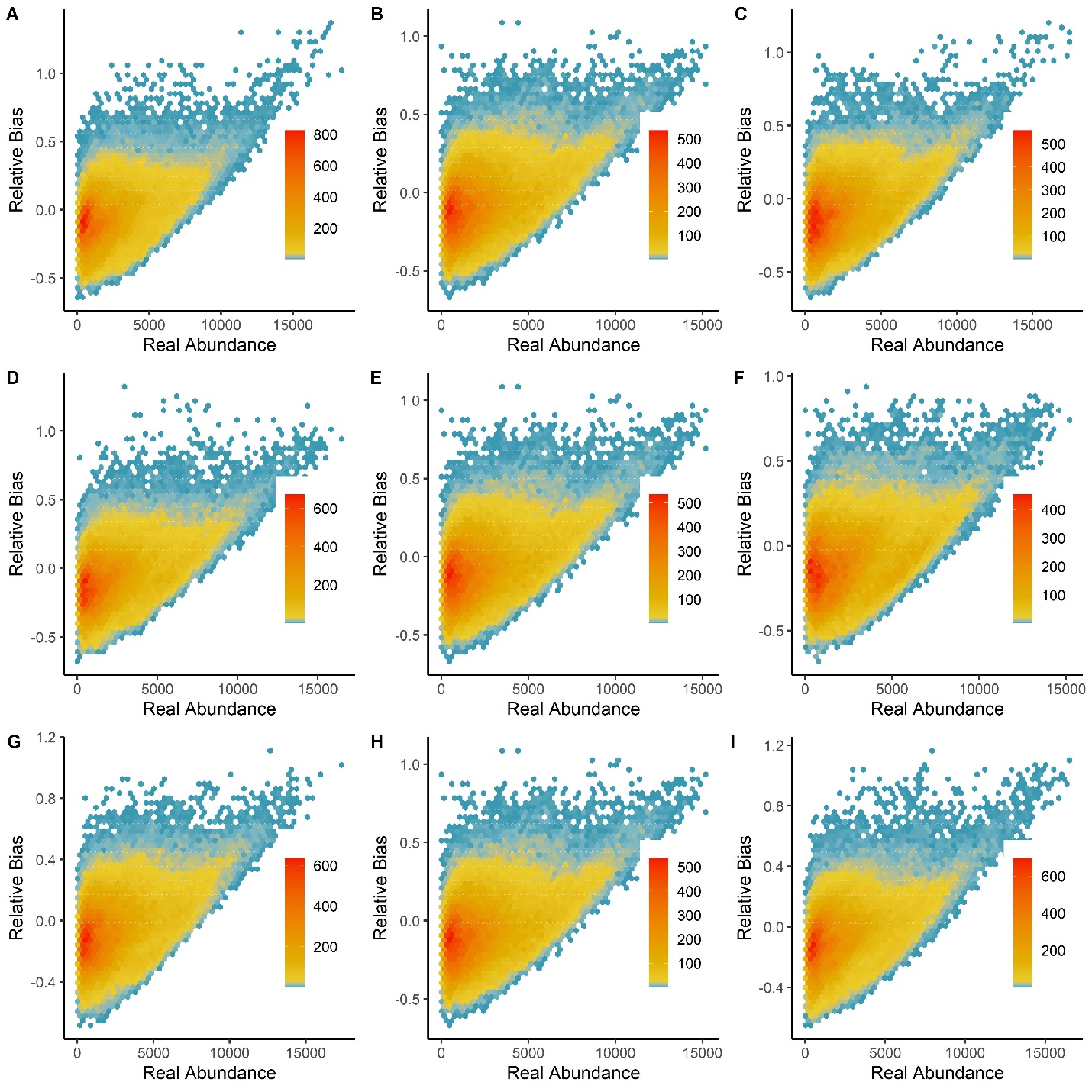


Figure S3. Relative bias in region specific abundance estimates were consistent regardless of the spatial configuration in harvest rate. Relationship between relative bias and real abundance is depicted for each simulation set, which differed accord to their magnitude of variation (A-C), the maximum distance of autocorrelation (D-F), and the amount of small scale variation (G-I) in harvest rate.


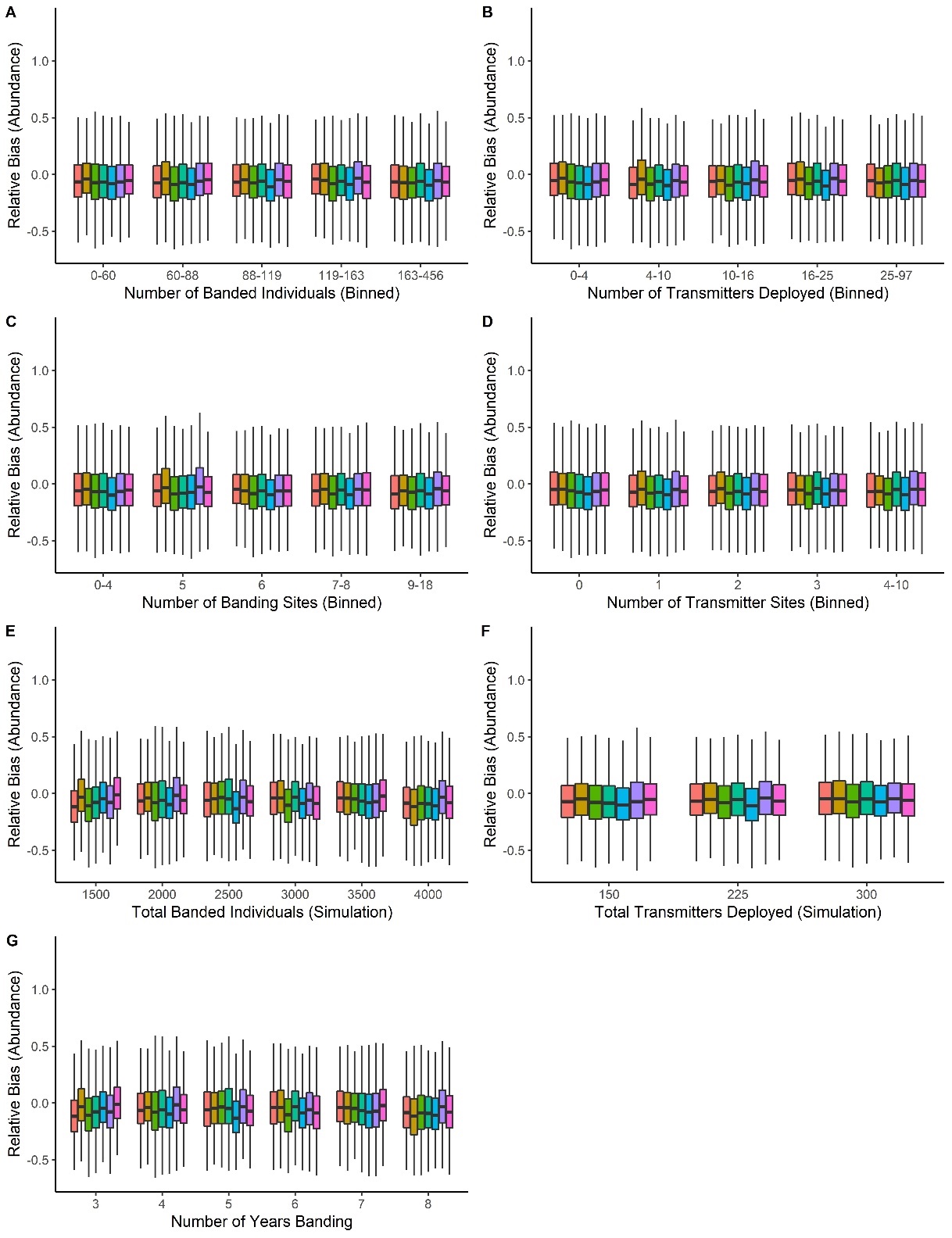


Figure S4. Relative bias in region specific estimates of abundance were consistent across variable sample sizes for different data inputs, illustrated here by a summary of simulation results showing variation in bias according to sample size (x-axis) across a variety of spatial configurations in harvest rate (boxplot color; Figure 3).


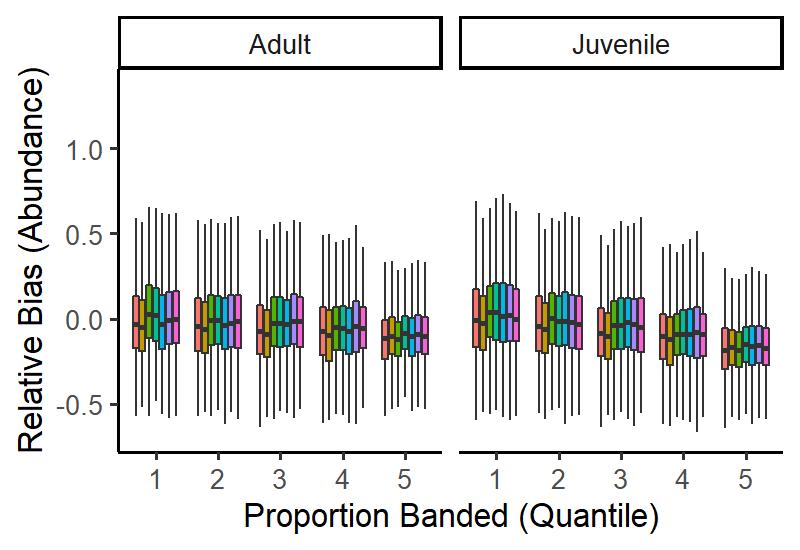


Figure S5. Relative bias in region-specific abundance estimates became more negative and less variable as the proportion of banded individuals in a population increased as evidenced by the summary of results from simulation data used in a Lincoln Estimator using a spatial predictive process to account for spatial variation. The model was tested under 7 different spatial configurations of variation in harvest rate (corresponding to color of boxplots).


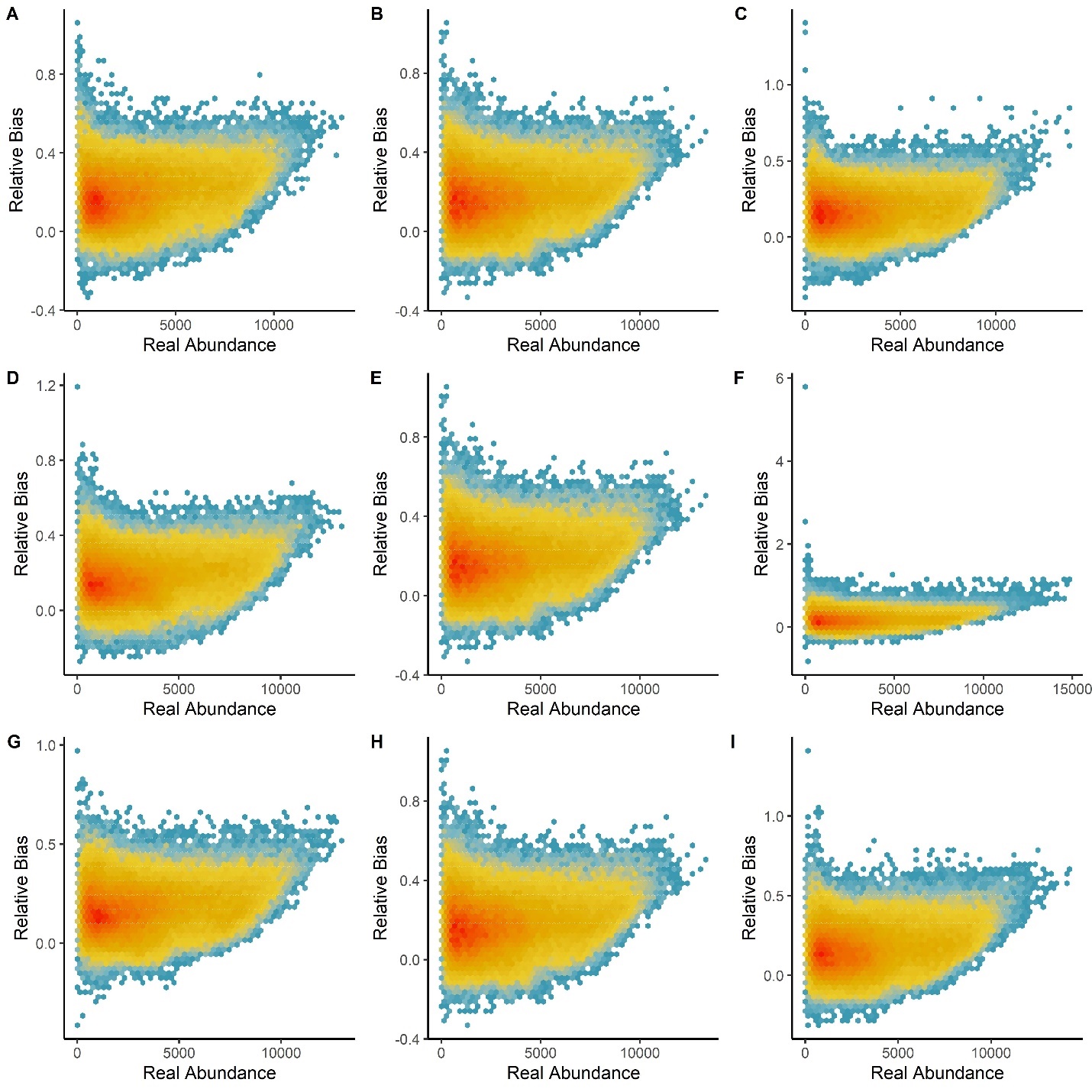


Figure S6. Regardless of the spatial configuration of harvest rates, relative bias was greater in region specific abundance estimates when a spatial predictive process was not included to account for heterogeneity. Relationship between relative bias and real abundance is depicted for each simulation set, which differed accord to their magnitude of variation (A-C), the maximum distance of autocorrelation (D-F), and the amount of small scale variation (G-I) in harvest rate.
